# Immune-Complexome Analysis Identifies Immunoglobulin-Bound Biomarkers That Predict the Response to Chemotherapy of Pancreatic Cancer Patients

**DOI:** 10.3390/cancers12030746

**Published:** 2020-03-21

**Authors:** Giorgia Mandili, Laura Follia, Giulio Ferrero, Hiroyuki Katayama, Wang Hong, Amin A. Momin, Michela Capello, Daniele Giordano, Rosella Spadi, Maria Antonietta Satolli, Andrea Evangelista, Samir M. Hanash, Francesca Cordero, Francesco Novelli

**Affiliations:** 1 Department of Molecular Biotechnology and Health Sciences, University of Turin, 10126 Torino, Italy; giorgia.mandili@unito.it (G.M.); laura.follia@unito.it (L.F.); 2 Center for Experimental Research and Medical Studies (CeRMS), University of Turin, 10126 Torino, Italy; 3 Department of Computer Science, University of Turin, 10149 Torino, Italy; giulio.ferrero@unito.it (G.F.); francesca.cordero@unito.it (F.C.); 4 Department of Clinical and Biological Sciences, San Luigi Hospital, University of Turin, 10043 Orbassano, Turin, Italy; 5 MD Anderson Cancer Center, University of Texas, Houston, TX 77030, USA; 6 Centro Oncologico Ematologico Subalpino (COES), University of Turin, 10126 Torino, Italy; 7 Cittá della salute e della scienza University Hospital of Turin, University of Turin, 10126 Torino, Italy

**Keywords:** proteomics, pancreatic cancer, chemotherapy, immune complexes, biomarkers, TAA, computational analysis

## Abstract

Pancreatic Ductal Adenocarcinoma (PDA) is an aggressive malignancy with a very poor outcome. Although chemotherapy (CT) treatment has poor efficacy, it can enhance tumor immunogenicity. Tumor-Associated Antigens (TAA) are self-proteins that are overexpressed in tumors that may induce antibody production and can be PDA theranostic targets. However, the prognostic value of TAA-antibody association as Circulating Immune Complexes (CIC) has not yet been elucidated, mainly due to the lack of techniques that lead to their identification. In this study, we show a novel method to separate IgG, IgM, and IgA CIC from sera to use them as prognostic biomarkers of CT response. The PDA Immune-Complexome (IC) was identified using a LTQ-Orbitrap mass spectrometer followed by computational analysis. The analysis of the IC of 37 PDA patients before and after CT revealed differential associated antigens (DAA) for each immunoglobulin class. Our method identified different PDA-specific CIC in patients that were associated with poor prognosis patients. Finally, CIC levels were significantly modified by CT suggesting that they can be used as effective prognostic biomarkers to follow CT response in PDA patients.

## 1. Introduction

The role of the immune system in cancer has been extensively studied, due to its critical role in counteracting tumor development. The field of cancer immunology has elicited growing interest because of the success obtained in many cancers [1]. Even in tumors defined as immunologically “cold“ (i.e., with a low infiltrate of T cells), such as pancreatic cancer, the possibility to exploit the immune system to identify biomarkers or immunological targets has been demonstrated [2,3,4,5,6,7].

Immune complexes (IC) are those formed between the antibody and its antigen [8]. The self- or non-self-antigens are bound to immunoglobulins by non-covalent interactions. Circulating Immune Complexes (CIC) are present in healthy subjects and cooperate in the immune response [9]. In healthy subjects, IC are removed from the bloodstream, while, in pathological conditions, CIC can accumulate in the bloodstream or in different tissues, especially in the kidneys, inducing glomerulonephritis [8]. Pathological CIC were discovered as biomarkers of Raynaud’s syndrome [8]. Indeed, the accumulation of CIC could provide a novel source of biomarkers for the diagnosis and the follow-up of a pathological condition. The use of CIC as disease biomarkers was specifically studied for autoimmune diseases, such as systemic lupus erythematosus [9,10] and rheumatoid arthritis [8], but it was also observed in other pathologies, including infectious diseases and acquired thrombotic thrombocytopenic purpura [8,11,12]. Of note, the level of CIC increases in the presence of several malignancies. In the 1970s, CIC were found in melanoma, osteogenic sarcoma, lymphoma and carcinomas of the colon, lungs, breast, prostate, pancreas, stomach, esophagus, and uterine cervix [13,14]. However, a systematic analysis of CIC was provided only in 2013 in uveitis, using pull-down assays to separate IgG-IC [15]. One of the main reasons for this delay was the lack of a specific assay to detect and quantify CIC, which led to the missing identification of specific biomarkers in malignancies until now.

With the advent of high-throughput technologies, such as mass spectrometry, the study of CIC has become more accurate. Thus, several techniques have been used to study IC, including ultracentrifugation, ELISA, gel filtration, ultrafiltration, precipitation with polyethylene glycol, assays based on interactions with purified complement portions, quantitative dot-blot assay, and Raji cell radioimmunoassay. Furthermore, CIC were also studied through assays that measured their effects on other cells such as platelet aggregation tests, inhibition of antibody-dependent cytotoxicity assays, tests for the release of enzymes from eosinophils and mast cells, and macrophages inhibition assays [8,9,10,13]. However, most of these techniques allow the investigation of the presence of CIC bounded with already known antigens, without identifying novel CIC-associated antigens.

Immunoglobulin (Ig) G is the main Ig class in human blood, and it represents about 70% of the total Ig pool. For this reason, in studies performed on the humoral response, attention has mainly been focused on IgG class response, including our previous studies on antibody response in PDA patients [2,4,16]. However, the role of other Ig classes in cancer could also be relevant. Indeed, it was demonstrated that IgM and IgG antibodies were bound to allogeneic tumor cells [17]. Conversely, a subpopulation of IgA-secreting B cells that suppresses the activation of CD8${}^{+}$ T cells in prostate cancer, after oxaliplatin treatment, was identified [18]. Moreover, several studies identified IgM CIC containing squamous cell carcinoma antigen or carcinoembryonic antigen, which have diagnostic value in hepatocellular carcinoma and colorectal cancer, respectively [19,20].

Demonstrating the association between CIC levels of cofilin-1 in sera with cancer progression and poor prognosis has highlighted the importance of IC in PDA [21]. It has been well established that chemotherapy (CT) influences the humoral and cellular immune responses to the tumors [22], and, therefore, identifying CIC that can predict responses or resistance to CT could allow the maximization of the efficacy of treatments and could avoid useless toxic effects in non-responding patients [23].

Here, we propose a systematic analysis of IgG, IgM, and IgA CIC in PDA. For the first time, we have demonstrated that, in PDA, identifying novel Tumor-Associated Antigens (TAA) associated with different classes of Ig patients treated with CT can provide new prognostic markers.

## 2. Results

**Figure 1:**
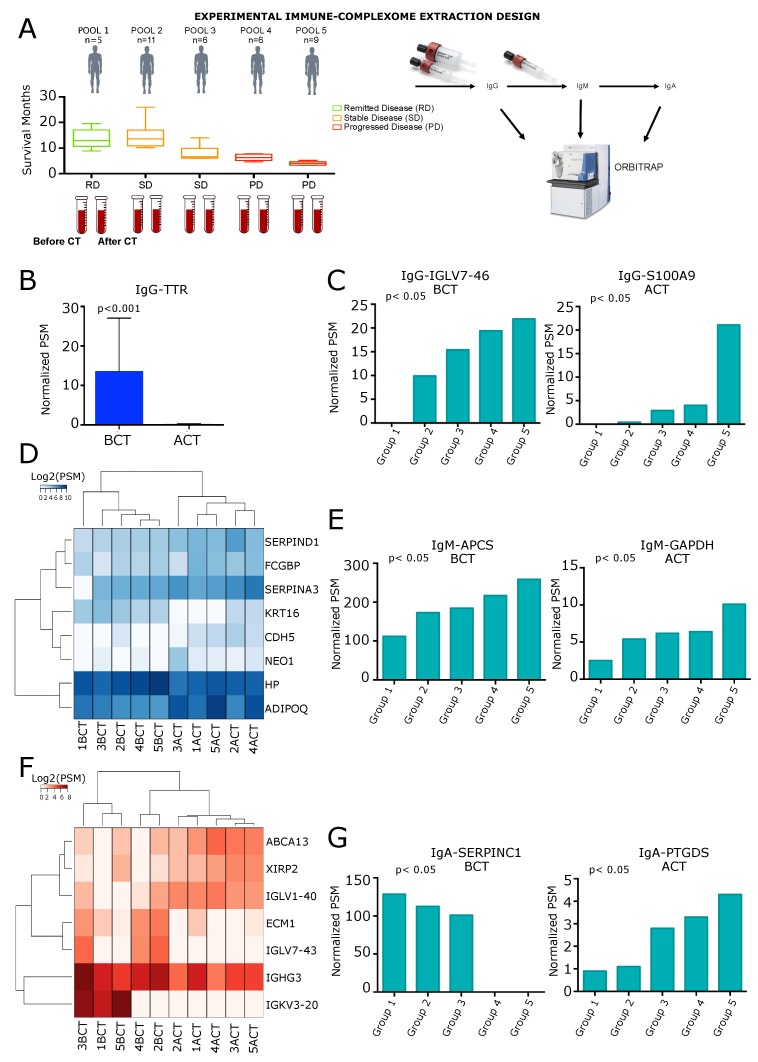
Analyses of IgG, IgM, and IgA immuno-complexes upon chemotherapy (CT) in sera of PDA patients. (**A**) scheme of the experimental design of the immune-complexome proteomic analysis using pulled sera from PDA patients collected before and after CT (BCT and ACT). Samples were divided into pools based on the months of disease-free survival; (**B**) Bar plot representing the circulating level of TTR (IgG differential associated antigen (DAA)) BCT and ACT; (**C**) bar plots representing the most significant trends of IgG Circulating Immune Complexes (CIC) considering BCT and ACT results separately. Both IgG-IGLV7-46 (left panel) and IgG-S100A9 (right panel) increased from good to bad prognosis in the five groups; (**D**) heat map showing IgM DAA in the five groups of patients; (**E**) bar plots representing the most significant trends of IgM CIC considering BCT and ACT results separately. Both IgM-APCS (left panel) and IgM-GAPDH (right panel) increased from good to bad prognosis in the five groups; (**F**) heat map showing IgA DAA in the five groups of patients; (**G**) bar plots representing the most significant trends of IgA CIC considering BCT and ACT results separately. IgA-SERPINC1 (left panel) decreased from good to bad prognosis and IgA-PTGDS (right panel) increased from good to bad prognosis in the five groups. All CIC are represented as number of Peptide-Spectrum Matches (PSM).

We performed a systematic analysis of the PDA IC. The CIC-containing different Ig subclasses were sequentially separated and then analyzed by high resolution mass spectrometry (further details in Section 4). Sera from PDA before and after gemcitabine-based CT (namely BCT and ACT, respectively) were collected and analyzed. The IC bound to IgG, IgM, and IgA classes obtained from 37 PDA patients were subdivided into five groups characterized by different disease prognosis. Group one consisted of patients with the best prognosis, while in group five were patients with the worst prognosis (Supplementary Table S1A and Figure 1A). In each group, sera were pooled and the pooled IC content was analyzed using an LTQ-Orbitrap mass spectrometer (Figure 1A). For detailed information, see the Materials and Methods section). In order to identify CIC markers that could be informative for PDA prognosis and CT response, we analyzed the pooled sera of each BCT and ACT group. In this analysis, we identified 3547 proteins bound to IgG, 1043 proteins bound to IgM, and 1143 proteins bound to IgA before and after CT (Supplementary Table S1B–D). For each class of immunoglobulins, we performed a three-step computational analysis. Firstly, to investigate the effect of CT on the CIC content, the presence of each antigen before and after CT in each group was analyzed. Then, the different levels of CIC measured in the five BCT and ACT groups were analyzed to identify possible trends (i.e., a CIC that is found to increase or decrease progressively from group one to group five). Finally, the ACT/BCT ratio for each CIC in each group of patients was considered.

### 2.1. Analysis of IgG-Circulating Immune Complexes

The analysis of IgG-bound proteins BCT and ACT revealed that only the TTR (thyroid hormone-binding protein) CIC was significantly down-modulated by gemcitabine treatment (Figure 1B, $p=9.64\times 10^{4}$, Table 1 and Supplementary Table S2A). Considering BCT and ACT results separately, the analysis of different CIC levels in the five cohorts showed a statistically significant trend for IGKV6D-21 (Immunoglobulin kappa variable 6D-21) and IGLV7-46 (Immunoglobulin lambda variable 7-46) CIC in BCT data (Table 2). Conversely, IgG CIC of APOC2 (Apolipoprotein C-II), C4B (Complement C4-B), and S100A9 (Protein S100-A9) increased significantly from group one to group five in ACT data (*p* = 0.0274 and *p* = 0.043, respectively) (Table 3). Interestingly, results from the trend analysis demonstrated that IGLV7-46 (BCT) and S100A9 (ACT) showed the most significant trend (*p* = 0.027) as they were highly abundant in the low survival patient group and absent in the high survival patient blood (Figure 1C, Supplementary Table S3A). Trend analysis of CIC level BCT w.r.t. ACT (ACT/BCT ratio) in the five groups revealed 79 differentially recognized proteins (Supplementary Table S4A and Table S5A), with IGHV4-34 (immunoglobulin heavy variable 4-34) displaying a progressively increasing abundant trend (ratio ACT/BCT: 1, 0.57, 0.07, 60.2, and 119.3 for groups 1, 2, 3, 4, and 5, respectively).

### 2.2. Analysis of IgM Circulating Immune Complexes

The analysis of IgM-bound proteins BCT and ACT revealed the statistically significant variation of eight different protein levels, namely ADIPOQ (adiponectin), CDH5 (Cadherin-5), FCGBP (IgGFc-binding protein), SERPINA3 (alpha-1-antichymotrypsin), NEO1 (Neogenin) and SERPIND1 (Heparin cofactor 2), which were increased ACT, and HP (Haptoglobin) and KRT16 (Keratin, type I cytoskeletal 16), which were decreased ACT (Figure 1D, Table 1 and Supplementary Table S2B). Analyzing the IgM CIC levels in the five cohorts, considering BCT and ACT separately, revealed that seven proteins were significantly modulated w.r.t. patient survival: AOC3 (membrane primary amine oxidase), APCS (serum amyloid P-component), APOB (apolipoprotein B-100) and IGLV3-25 (immunoglobulin lambda variable 3-25) in the BCT data (Table 2). GAPDH (glyceraldehyde-3-phosphate dehydrogenase), IGLV1-47 (immunoglobulin lambda variable 1-47), and OLFM2 (noelin-2) were increased significantly in the ACT data (Table 3). CIC containing AOC3, APOB, IGLV3-25, IGLV1-47, and OLFM2 decreased from group 1 to group 5 while those with APCS and GAPDH increased in abundance (Figure 1E, Table 2 and Table 3 and Supplementary Table S3B). Comparing the ACT/BCT ratios between the five groups, 30 proteins were identified as being significantly modulated (Supplementary Tables S4B and S5B). Of such proteins, ADIPOQ, AOC3, and SERPINA1 showed the most significant trends (*p*-value < 0.0001), and their level progressively increased from group 1 to group 5 (Supplementary Table S4B).

### 2.3. Analysis of IgA Circulating Immune Complexes

The analysis of IgA-bound proteins BCT and ACT revealed the significant modulation of seven proteins: ABCA13 (ATP Binding Cassette Subfamily A Member 13), IGLV1-40 (Immunoglobulin Lambda Variable 1-40) and XIRP2 (Xin Actin Binding Repeat Containing 2) which increased ACT and ECM1 (Extracellular Matrix Protein 1), IGHG3 (Immunoglobulin Heavy Constant Gamma 3), IGKV3-20 (Immunoglobulin Kappa Variable 3-20) and IGLV7-43 (Immunoglobulin Lambda Variable 7-43) which decreased ACT (Figure 1F, Table 3 and Supplementary Table S2C). Considering results BCT and ACT separately, the analysis of CIC levels in the five cohorts showed a statistically significant trend for 10 proteins: APOL1 (apolipoprotein L1), IGHV3OR16-9 (immunoglobulin heavy variable 3/OR16-9), MMRN2 (multimerin-2), SERPINC1 (antithrombin-III), and SERPIND1 (heparin cofactor 2) in the BCT condition (Table 2), while C9 (complement component C9), FREM2 (FRAS1-related extracellular matrix protein 2), IGHD (immunoglobulin heavy constant delta), KCNQ2 (potassium voltage-gated channel subfamily KQT member 2), and PTGDS (prostaglandin-H2 D-isomerase) were detected in the ACT condition (Table 3 and Supplementary Table S3C). Interestingly, all these CIC, increased from group 1 to group 5, except for SERPINC1, which decreased in abundance (Figure 1G and Table 2). In ACT CIC containing PTGDS progressively decreased from group 1 to group 5 (Figure 1G and Table 3). Comparing the modulation of CT in the trends of CIC BCT and ACT in the five groups, 47 proteins were identified as statistically significantly related to patient survival (Supplementary Tables S4C and S5C). PTGDS, KCNQ2, PDE4A, MAN1C1, IGHV3-23, OBSNC, and BRSK2 were characterized as having the most significant trend that increased from group 1 to group 5 while MRPS26 decreased significantly from group 1 to group 5.

### 2.4. Enrichment Analysis of IgA, IgM and IgG CIC

To predict candidate biological processes and signaling pathways that are enriched in the set of antigens bound to the CIC, we performed an enrichment analysis of combined IgA, IgM, and IgG DAA. This revealed different enriched processes and phenotypes including “PDA genesis“, “type II diabetes mellitus onset“, and “extracellular vesicles“ (Supplementary Table S6). PDA genesis, insulin secretion, and diabetes pathways confirm the accuracy of our analysis.

## 3. Discussion

The relationship between CIC and tumors has been known for many years, but a full and complete comprehension of the role of CIC in cancer is still lacking. The observation of elevated levels of CIC in the presence of malignancies dates back to the 1970s [13,14]. However, the study of CIC remained a challenge until the development of mass spectrometry. In fact, in the past, CIC were studied with methods that separated them only roughly, and assumed for the analysis the prior knowledge of the antigen under study, thus not allowing the discovery of new antigens [8,9,10,13]. Instead, the combination of a proper CIC separation and mass spectrometry as presented here leads to the identification of the proteins involved in the disease, opening new perspectives to investigate the role of these CIC in cancer.

In this article, we have proposed a novel approach to investigate CIC that has led us to identifying important TAA suitable to be follow-up biomarkers. The analysis was performed on the sera of patients with PDA before and after gemcitabine-based CT. Currently, we are recruiting a large number of PDA patients in a multi-center clinical study in order to confirm and validate the prognostic role of CIC-bound TAA identified in this study.

In contrast to previously used methods that allowed the separation of all immunoglobulin repertoire (ultracentrifugation, gel filtration, ultrafiltration, precipitation with polyethylene glycol) [8] or the enrichment of CIC containing IgG only [15,16], we presented a systematic analysis of CIC containing IgG, IgM, and IgA isotypes that we define as the “immune-complexome“. This represents a technical and conceptual improvement that was found of particular interest. In fact, we demonstrated that the proteins bound to different classes of immunoglobulins, and their changes in relation to CT, defined different patterns of response to CT, underlying the need to consider them separately. Of interest, proteins bounded to immunoglobulins also showed marked differences with respect to serum-free circulating proteins [24].

The most interesting outcome of this study is that the clinically relevant CIC trends were from the IgM fraction. It is known that IgM CIC are more frequent w.r.t. IgG ones because the clearance of IgG CIC is maintained by (i) the activation of complement by a classical pathway and (ii) the Fc gamma receptors (FcγR) system which is present on the surface of different cells of the immune system. This second modality of CIC clearance is not employed for IgM CIC. For this reason, it is possible that proteins bound to IgM reflect the earliest humoral response to PDA after CT. Of note, the IgG fraction is not the most important class associated with protein markers in our analysis; although sera IgG were the most present quantitatively, they showed less informative cues when the purpose was to identify of IgG-associated biomarkers following CT treatment.

Enrichment analysis strongly reinforced the robustness of our study, highlighting many processes related to pancreatic cancer (PDA genesis, type II diabetes mellitus onset, and insulin secretion). Notably, the enrichment in protein localized in the extracellular vesicle was particularly interesting, since a significant enrichment of exosome in the Ig-bound fraction was demonstrated in the sera of PDA patients compared to healthy subjects [16]. Moreover, several identified antigens have been previously correlated to PDA, although not in association with immunoglobulins, namely TTR (thyroid hormone-binding protein) and S100A9 (protein S100-A9) were found in IgG CIC [25,26], ADIPOQ (adiponectin), CDH5 (cadherin-5), FCGBP (IgGFc-binding protein), HP (haptoglobin), GAPDH (glyceraldehyde-3-phosphate dehydrogenase), and SERPINA1 (serine proteinase inhibitor A1) in IgM CIC [24,27,28,29], BRSK2 (BR Serine/Threonine-Protein Kinase 2) in IgA CIC.

TTR and HP bound to fucose have been already proposed as a biomarkers of PDA [30,31]; CDH5 in pancreatic neuroendocrine tumors developed in Von Hippel–Lindau disease patients [32] and FCGBP in mucinous cysts in the pancreas [33] and PDA [34]. S100A9 is a calcium- and zinc-binding protein with a key function in regulating inflammation and immune responses, and has been previously associated with PDA inflammation status, invasiveness, and metastasis [25,35]; in our experimental setting, S100A9 levels increased in case of worst prognosis. ADIPOQ levels were correlated to various kinds of cancers [36], but its role has not been well understood, yet. Indeed, even though the preponderance of evidence suggests an inverse correlation of serum ADIPOQ levels with cancer, other studies correlated the increase of ADIPOQ levels with cancer progression [36]. In the case of PDA, the scenario is particularly disputable. Adiponectin inhibits PDA proliferation of Pan02 cells both in vitro and in vivo when cells were transplanted in a murine model, by induction of caspase-3 and caspase-7 and apoptosis [37]. Conversely, in the same year, it was showed that ADIPOQ inhibited apoptosis of pancreatic cancer cells in the same murine model via activation of AMPK-Sirt1-PGC1α signaling [27]. In human studies, the situation is controversial; adiponectin in pancreatic cancer patients has been separately observed to be elevated [38,39,40], decreased [41,42], and unchanged [43,44]. A possible explanation of these conflicting results could be that, as shown in this study, ADIPOQ is not important by itself, but the ADIPOQ bound to IgM. In fact, we showed a very interesting trend, with IgM-ADIPOQ levels significantly increasing with worse prognoses.

GAPDH, which we observed to increase in being bound to IgM from the group one to the group five ACT, has already been correlated to PDA with a worse prognosis [24]. SERPINA1 is up-regulated in PDA tissue and serum [45] and has been proposed as a prognostic factor in pancreatic cancer [46]. BRSK2, which we saw to increase from group one to group five, was demonstrated to provide a survival advantage to PDA cells [47].

Other identified antigens have been correlated to other types of cancer, namely ABCA13 in ovarian cancer [48], XIRP2 in gastric cancer [49], and APOC2 expression in Non-Small Cell Lung Cancer [50]. Typical serum proteins were among the identified ones, despite the immune depletion before the mass spectrometry analysis. We found complement proteins, immunoglobulins, apolipoproteins, and inflammation proteins, some of which were significantly modulated by CT, namely APOC2 (apolipoprotein C-II), C4B (complement C4-B), IGLV7-46, IGHV4-34 (immunoglobulin heavy variable 4-34) in IgG CIC, APOB (apolipoprotein B-100), IGLV3-25 (immunoglobulin lambda variable 3-25), IGLV1-47 (immunoglobulin lambda variable 1-47) and SERPINA1 in IgM CIC, APOL1 (apolipoprotein L1), C9 (complement component C9), IGHG3, IGKV3-20, IGLV1-40, IGLV7-43, IGHV3OR16-9 (immunoglobulin heavy variable 3/OR16-9), IGHD (immunoglobulin heavy constant delta), IGHV3-23, SERPINC1 (antithrombin-III) and SERPIND1 (heparin cofactor 2) in IgA CIC. Concerning the role of CIC in cancer, very little data are available. It has been reported that proteins bound to Ig could have a decoy function, inhibiting antibody-dependent cytotoxicity [16]. This aspect could be relevant to explain the increase of the majority of the identified CIC after CT in parallel with prognosis getting worst; however, it is certainly not the only role. It is likely that CIC binding could enhance the immune response against a protein, but it could also sequester a protein rendering it ineffective. Depending on the role of the antigen, this behavior could lead to a good or bad prognosis.

## 4. Materials and Methods

### 4.1. Patients

From 2006 to 2012, 37 patients with PDA, enrolled in ENOAPA project (https://www.epiclin.it/enoapa), provided serum samples. The protocol was approved by the local research ethical committee (Azienda Ospedaliera Città della Salute e della Scienza di Torino, Turin) ethical code #0114454, November 23th, 2016.) and was performed according to the Helsinki Declaration principles. All participants in the protocol signed a declaration of informed consent. No patients were subjected to surgery and all were treated with gemcitabine-based CT (gemcitabine with oxaliplatin or alone). Serum samples were isolated from venous blood before and after CT and stored at −80 ${}^{\circ }$C until use. Patients’ characteristics are listed in Supplementary Table S1A. Patients were grouped into five groups on the basis of response to therapy and survival, as indicated in Supplementary Table S2. An equal amount of serum from each patient in each group was taken to reach a total volume of 300 μL.

### 4.2. IgG-IC Purification

HiTrap Protein G HP (1 mL) was used (GE HealthCare Life Sciences, Milan, Italy). The column was operated with a peristaltic pump. All buffers were filtered before use. Serum was filtered with a SPARTAN Syringe Filter, RC Filter Media, 13 mm diameter, 0.2 μm pore size (Whatman, Maidstone, UK) and then diluted at 1:3 in binding buffer (20 mM sodium phosphate, pH 7.0). The column was washed with 5 mL of milliQ water and with 5 mL of binding buffer before sample application. The sample was then passed through the column along with 9 mL of binding buffer, collecting the “unbound fraction“ exiting from the column. IgG-IC were eluted from the column with 10 mL of elution buffer (0.1 M glycine-HCl, pH 2.7) and collected in a tube containing 1 mL of neutralizing buffer (1 M Tris-HCl, pH 9.0). All timing and volumes were optimized evaluating IgG presence by Western blotting.

### 4.3. IgM-IC Purification

After the evaluation of their presence by Western blotting, IgM-IC were separated from the IgG unbound fraction by HiTrap IgM purification HP 1 mL (GE HealthCare Life Sciences, Milan, Italy). The column was operated with a peristaltic pump. The sample should have the same concentration of ammonium sulfate as the binding buffer, so small amounts of solid ammonium sulfate were gradually added to the sample until the final concentration was 1M, stirring slowly and continuously, to avoid IgM precipitation. Samples were then filtered with SPARTAN Syringe Filter, RC Filter Media, 13 mm diameter, 0.2 μm pore size (Whatman, Maidstone, UK). All buffers were filtered before use. Columns were washed with 5 mL of binding buffer (20 mM sodium phosphate, 1 M ammonium, pH 7.5), 5 mL of elution buffer (20 mM sodium phosphate, pH 7.5) and 5 mL of regeneration buffer (20 mM sodium phosphate, pH 7.5 with 30% isopropanol) and then equilibrated with 5 mL of binding buffer. This was then applied to the sample. The unbound fraction was washed out with 15 mL of binding buffer. Elution was performed with 12 mL of elution buffer. Columns were then regenerated with regeneration buffer and equilibrated with a binding buffer for the subsequent separation. All timings and volumes were optimized evaluating IgM presence by Western blotting.

### 4.4. IgA-IC Separation

IgA-IC were present with the eluted IgM-IC fraction, as verified by Western blotting. Indeed, starting from the elution containing IgM-IC, IgA-IC were separated by immunoprecipitation with anti-human IgA (a chain specific)-agarose antibody (Sigma Aldrich, St. Louis, MO, USA). After adding 1.9 mL of antibody, samples were incubated overnight at 4 ${}^{\circ }$C on a rocking platform. After three washes with PBS, IgA-IC were eluted with two elutions of 4 mL each of elution buffer (0.1 M glycine-HCl, pH 2.7). Elutions were collected in a tube containing 0.8 mL of neutralizing buffer (1 M Tris-HCl, pH 9.0).

### 4.5. LC-MS Analysis

All samples were concentrated to a volume of 500 μL using a 3KDa cut-off concentrator (Merck Millipore, Burlington, MA, USA) at 4000 *g* for 60 min. They were then reduced with TCEP (35 μL in each sample; incubation at 37 ${}^{\circ }$C for 30 min) and alkylated with acrylamide (20 mg in each sample; incubation at room temperature for 30 min in the dark). After buffer exchange with a solution containing 20 mM Tris, 4 M urea, 3% isopropanol, pH 8.0, samples were subjected to reverse phase separation as previously described [51]. Samples were then fractionated in protein level using the Shimadzu HPLC system. The protein sample was loaded onto the RPGS reversed-phase column (4.6 mm I.D. ×150 mm, 15 μm, 1000Å, Column Technology Inc., #RP5D46150) and desalted for 5 min with 95% mobile-phase A (0.1% TFA in 95% H${}_{2}$O) at a flow rate of 3 mL/min. Proteins were eluted from the column at a flow rate of 2.1 mL/min with a gradient elution including: (i) an increase from 5 to 70% mobile phase B (0.1% TFA in 95% ACN) for more than 25 min, (ii) an increase from 70% to 95% mobile phase B for 3 min, (iii) a wash step to hold at 95% mobile phase B for 2 min, (iv) a re-equilibration step at 95% mobile phase A for 5 min. The collected fractions were pooled into 15 fractions. All fractions obtained in this way were lyophilized and proteins were digested with trypsin. The analysis was performed using Orbitrap Elite. The tryptic peptides were separated by reversed-phase chromatography using an EASYnano HPLC system (Thermo Scientific, Waltham, MA, USA) coupled online with a LTQ-Orbitrap ELITE mass spectrometer (Thermo Scientific, Waltham, MA, USA). Mass spectrometer parameters were: spray voltage 2.5 kV, capillary temperature 280 ${}^{\circ }$C, Furier Transform (FT) resolution 60,000, FT target value 1 ×$10^{6}$, LTQ target value 3 ×$10^{4}$, 1 FT microscan with 500 ms injection time, and 1 LTQ microscan with 10 ms injection time. Mass spectra were acquired in a data-dependent mode with the m/z range of 400–2000. The full mass spectrum (MS scan) was acquired by the FT, while the tandem mass spectrum (MS/MS scan) was acquired by the LTQ with 35% normalized collision energy. Acquisition of each full mass spectrum was followed by the acquisition of MS/MS spectra for the five most intense +2 or +3 ions within a one second duty cycle. The minimum signal threshold (counts) for a precursor occurring during a MS scan was set at 1000 for triggering an MS/MS scan.

The acquired LC-MS/MS data were processed by the Proteome Discoverer 1.4 (Thermo Scientific, Waltham, MA, USA). Sequest HT was used as a search engine with input parameters including cysteine (Cys) alkylated with acrylamide (71.03714@C) as a fixed modification and methionine (Met) oxidation (15.99491@M) as a variable modification. Data were searched against the Uniprot Human database 2017 and further filtered with a False Discovery Rate (FDR) <=5%. Total mass spectra counts for each protein were used as a measure of protein abundance.

### 4.6. Protein Identification and Bioinformatics Aanalysis

A new R computational pipeline to pre-process, normalize, and analyze obtained proteomic data was applied. Our pipeline consisted of data characterization, individuation of differentially associated antigen (DAA), trend analysis, and functional enrichment analysis. Using our approach, we identified DAA and their trends upon CT. We also characterized the enriched biological processes and phenotypes of the antigens identified.

### 4.7. Data Pre-Processing and Normalization

Spectra obtained from LTQ-Orbitrap were analyzed using SEQUEST software. SEQUEST identified peptide sequences from tandem mass spectra. Peptides-Spectrum Matches (PSMs) obtained from this analysis were filtered in order to take into consideration only IC with PSM > 1 or IC with PSM equal to 1 occurring in at least two groups. Filtered PSMs were normalized using the quantile normalization method in R.

### 4.8. Differential Associated Antigens and Antigen Recognition Trends

Comparisons of IC abundance (before and after CT) were computed using the DESeq2 R package [52]. Analysis of IC abundances’ trends among the five sample pools was performed using MaSigPro R package [53] with default parameters.

### 4.9. Gene Set Enrichment Analysis

Gene list coding for identified proteins was retrieved using UniProt and BioDBnet and analyzed using an Enrichr tool [54].

## 5. Conclusions

In conclusion, our method leads: i) to the identification of novel PDA specific CIC before and after gemcitabine treatment, ii) to the characterization of CT effects on CIC levels. In this study, we developed a new method that combines the capture of IC and mass spectrometry, allowing the discovery that CIC-bound TAAs could be markers of CT response. Some of the identified candidate CIC could be suitable for use as novel non-invasive prognostic markers.

## Figures and Tables

**Table 1:** List of differentially recognized Circulating Immune Complexes.

Gene Name	Ig Subtype	Variation Following Chemotherapy
TTR	IgG	decreased
*ADIPOQ*	IgM	increased
*CDH5*	IgM	increased
*FCGBP*	IgM	increased
*HP*	IgM	decreased
*KRT16*	IgM	decreased
*NEO1*	IgM	increased
*SERPINA3*	IgM	increased
*SERPIND1*	IgM	increased
*ABCA13*	IgA	increased
*ECM1*	IgA	decreased
*IGHG3*	IgA	decreased
*IGKV3-20*	IgA	decreased
*IGLV1-40*	IgA	increased
*IGLV7-43*	IgA	decreased
*XIRP2*	IgA	increased

**Table 2:** List of significant Circulating Immune Complexes trends before chemotherapy.

Gene Name	Ig Subtype	Trend (from Group 1 to 5)
*IGKV6D-21*	IgG	increasing
*IGLV7-46*	IgG	increasing
*AOC3*	IgM	decreasing
*APCS*	IgM	increasing
*APOB*	IgM	decreasing
*IGLV3-25*	IgM	decreasing
*APOL1*	IgA	increasing
*IGHV3OR16-9*	IgA	increasing
*MMRN2*	IgA	increasing
*SERPINC1*	IgA	decreasing
*SERPIND1*	IgA	increasing

**Table 3:** List of significant Circulating Immune Complexes trends after chemotherapy.

Gene Name	Ig Subtype	Trend (from Groups 1 to 5)
*APOC2*	IgG	decreasing
*C4B*	IgG	decreasing
*S100A9*	IgG	increasing
*GAPDH*	IgM	increasing
*IGLV1-47*	IgM	decreasing
*OLFM2*	IgM	decreasing
*C9*	IgA	increasing
*FREM2*	IgA	increasing
*IGHD*	IgA	increasing
*KCNQ2*	IgA	increasing
*PTGDS*	IgA	increasing

## Supplementary Materials

The following are available online at https://www.mdpi.com/2072-6694/12/3/746/s1, Table S1: Clinical features of patients and list of identified proteins. A. Table containing the clinical information of patients enrolled in the study B. List of proteins bound to IgG identified in the study C. List of proteins bound to IgM identified in the study D. List of proteins bound to IgA identified in the study, Table S2: Differential recognized antigens. A. List of IgG antigens significantly modulated by gemcitabine B. List of IgM antigens significantly modulated by gemcitabine C. List of IgA antigens significantly modulated by gemcitabine, Table S3: Significant Trends before and after CT. A. List of IgG CICs trends BCT and ACT with *p* < 0.05 B. List of IgM CICs trends BCT and ACT with *p* < 0.05 C. List of IgA CICs trends BCT and ACT with *p* < 0.05, Table S4: MaSig Pro Results of significant trends upon CT treatment. A. IgG significant trends computed by MaSig Pro. Only antigens recognized by at least three out of five groups of patients are represented. B. IgM significant trends computed by MaSig Pro. Only antigens recognized by at least three out of five groups of patients are represented. C. IgA significant trends computed by MaSig Pro. Only antigens recognized by at least three out of five groups of patients are represented, Table S5: ACT/BCT ratios of the different groups of patients. A. IgG ACT/BCT ratio of antigens with significant trends computed with MaSigPro. B. IgM ACT/BCT ratio of antigens with significant trends computed with MaSigPro. C. IgA ACT/BCT ratio of antigens with significant trends computed with MaSigPro, Table S6: EnrichR results. A. Analysis of combined IgA, IgM and IgG differential recognized antigens.

## Abbreviations

The following abbreviations are used in this manuscript:

PDA	Pancreatic Ductal Adenocarcinoma
CT	ChemoTherapy
IC	Immune-Complexome
CIC	Circulating Immune Complexes
DAA	Differential Associated Antigens
TAA	Tumor Associated Antigens
